# Effect of Tai Chi Training on Plantar Loads during Walking in Individuals with Knee Osteoarthritis

**DOI:** 10.1155/2020/3096237

**Published:** 2020-03-05

**Authors:** Zhiwang Zhang, Lingyan Huang, Yu Liu, Lin Wang

**Affiliations:** School of Kinesiology, Shanghai University of Sport, Shanghai, China

## Abstract

Tai Chi is an available method for the treatment of knee osteoarthritis (KOA). The impacts of Tai Chi on plantar loads of individuals with KOA are not fully understood. 46 participants with knee osteoarthritis were randomly assigned into the Tai Chi group (*n* = 23) or the control group (*n* = 23). The Tai Chi group attended a 6-month Tai Chi program, and the control group participated in a wellness education program. Novel Pedar-X system was used to collect the peak pressure (PP) and maximum force (MF) during walking before and 6 months after the intervention. Significant higher peak pressure and maximum force were observed in the 4th and 5th metatarsophalangeal joints in the Tai Chi group. However, there were significant declines in the peak pressure of the whole foot and the 2nd and 3rd metatarsophalangeal joints and maximum force of the heel in the control group. These results suggested that individuals with KOA might change the pattern of plantar loads during walking through Tai Chi, and plantar loads would be useful as a parameter to assess the effect of Tai Chi on knee osteoarthritis. This trial is registered with Clinical Trials: CHiCTR-TRC-13003264.

## 1. Introduction

Knee osteoarthritis (KOA) is a chronic musculoskeletal disorder resulting in pain, disability, and decreased quality of life [1, 2]. More than 70% of population aged 65 years or older suffers from symptomatic KOA [3]. The overall costs spent on treating KOA cause a large economic burden on individuals [4]. Females are more likely to suffer from KOA than males [5, 6].

Nonpharmacological and nonsurgical interventions can reduce pain and improve physical function in patients with knee OA [7]. Some previous studies show the effectiveness of some interventions such as traditional Chinese medicine (auriculotherapy, acupuncture) and whole body vibration [8], orthotic devises [9], and electrical stimulation [10]. In addition, a systematic review of guidelines for the management of osteoarthritis proposes that exercise is a key factor for treating OA [11].

Exercise and physical therapies are recommended for nonpharmacological management of KOA [12–14]. Thus, selecting an appropriate form of exercise and physical therapies for females with KOA is vital in treating such condition.

The feet are the only segment of the human body that contacts the external environment directly during walking. Foot plantar load is the pressure field that acts between the support surface and the foot during walking [15]. The typical applications of plantar loads are injury prevention [16], footwear design [17], and sport performance [18]. Some studies [19, 20] had revealed that the ankle may play a compensatory role in individuals with KOA to relieve the pain or discomfort during walking, and loading patterns in the feet were related to KOA [21]. Zhang et al. [22] found that peak pressure and maximum force were significantly different from KOA females in midfoot and forefoot when compared with normal females. Rosland et al. [23] also reveal that maximum force in plantar distribution in individuals with KOA was associated with pain intensity, function, specific pain mechanisms, and radiological findings. Therefore, the management of plantar loads should be a part in the treatment of KOA.

Tai Chi, which is low impact and aerobic, is a form of mind–body therapy [24]. Given that Tai Chi includes many fundamental postures that flow smoothly from one to another [25], it can be an appropriate therapy for old-aged people. Tai Chi is an effective management for individuals with KOA because it can reduce pain and promote muscle endurance [26], motor control, and postural stability [12].

However, most previous biomechanical studies [27, 28] of Tai Chi only focus on pain, physical function, kinematics, and kinetics of the knee joint for KOA, rarely considering plantar loads. Hence, the present study is aimed at investigating the effect of Tai Chi on plantar loads in individuals with KOA after a 6-month Tai Chi exercise program. We hypothesized that participants receiving Tai Chi will show larger plantar loads than participants treated with wellness education.

## 2. Materials and Methods

### 2.1. Trial Design

The study was a single-blind randomized trial that investigated the effects of 6-month Tai Chi exercise on plantar loads among individuals with KOA. The Chinese Clinical Trial Registry was CHiCTR-TRC-13003264, and the data of registration was 27/05/2013. The study was approved by the Ethics Committee of Shanghai University of Sport, and the approval document number was 2013-001.

### 2.2. Participant

Female individuals aged 60–90 years who met the American College of Rheumatology criteria were recruited from three local communities in Yangpu District, Shanghai. The American College of Rheumatology criteria to make a definite diagnosis of KOA is that individuals show pain in the knee plus any three of the following six factors: (1) age more than 50 y, (2) less than 30 min of morning stiffness, (3) presence of crepitus on active motion, (4) bony overgrowth, (5) bony tenderness, and (6) no palpable warmth of synovium [29].

**Figure 1:**
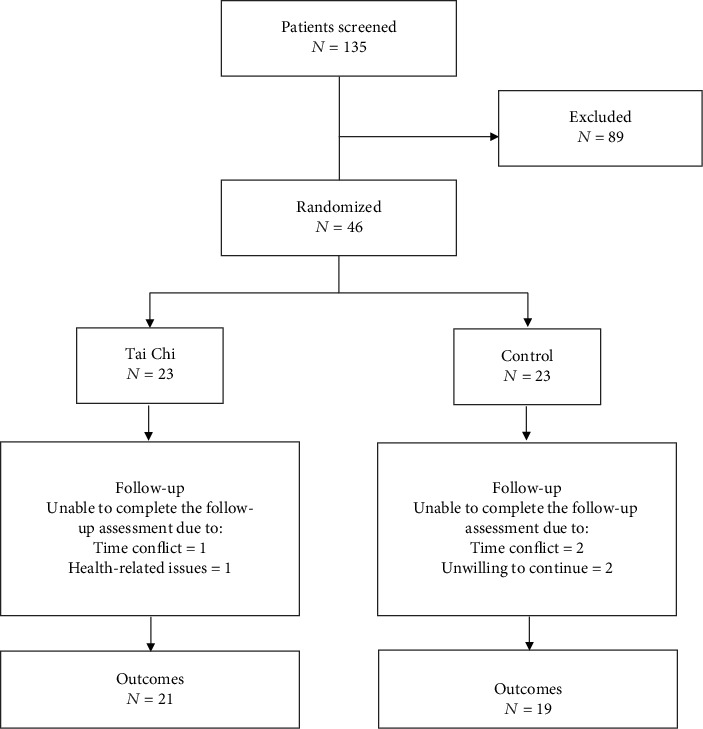
Flowchart explaining assignment of the participants to the Tai Chi and control groups.

The recruitment began in April 2013, and the baseline assessments were completed in June. The participants with mild to moderate KOA diagnosed by X-ray, pain symptoms for at least 12 weeks, and available for Tai Chi training or health education were included in the study [30]. Participants with surgery planned in the next 6 months, uncontrolled hypertension, cardiovascular diseases, and other illnesses that may affect their walking were excluded. Then, eligible participants were randomly assigned to either the Tai Chi group or the control group by computer. These two groups were blinded with each other. This study had obtained the patients' written informed consents to publish experimental results. The flow diagram of the trial is shown in Figure 1. All work was completed at the Shanghai University of Sport and the Shanghai Shangti Orthopedic Hospital.

### 2.3. Sample Size

The study was aimed at assessing the efficacy of Tai Chi intervention on plantar loads for women with knee OA. Participation by 17 participants per group (34 total) would provide 80% power to detect an effect size of 0.8 using a two-sided *t*-test with alpha = 0.05. Anticipating a 20% dropout rate, 20 participants should be enrolled for each group.

### 2.4. Interventions

The Tai Chi exercise, which was taught to the participants, was described in a previous study [30]. There were five Tai Chi movements, named brushing knee and twist steps, playing the lute, stepping back to repulse monkey, grasping sparrow's tail, and waving hands like clouds. In the first four weeks of the Tai Chi intervention, the participant was taught Tai Chi for two times per week. Each session included a 10-minute warm-up, learning new movements for 20 minutes, reviewing the learnt movements for 20 minutes, and 10-minute cooling down. In subsequent weeks, the Tai Chi program included a 10-minute warm-up, a 45-minute Tai Chi exercise, and cooling down for 5 minutes. The warm-up and cooling down sections included smooth breathing and gentle stretching of upper limb joints, lower limbs joints, and the trunk. Individuals should have no resistance to move their bodies after the warm-up and become relaxed after the cooling down. Two Tai Chi masters who had 15-year training experience were responsible for the instruction of Tai Chi exercise, and they were blinded to the randomization. The control group participated in a wellness education program. In the first session, research staff explained the aim of the program and procedures of the intervention. A variety of health professionals provided information which included diet and nutrition and mental and physical education (recognizing and dealing with stress and depression) in the following sessions; each session lasted for 60 minutes and was performed once a week for 6 months. This approach was successfully used in other studies [25, 31]. All subjects were encouraged to maintain their usual medication and ways of life.

### 2.5. Instrument and Measures

**Figure 2:**
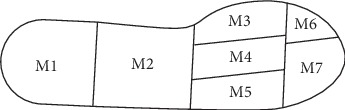
Foot mask. The foot was divided into 7 regions: heel (M1), midfoot (M2), the first metatarsophalangeal joint (M3), the 2nd and 3rd metatarsophalangeal joints (M4), the 4th and 5th metatarsophalangeal joints (M5), hallux (M6), and lessor toes (M7).

Peak pressure (PP) and maximum force (MF) were measured using the Pedar-X system (Novel GmbH, Munich Germany), sampling at 50 Hz. With the aid of the Trublu calibration device, all insoles of the Pedar-X system were calibrated before plantar loading assessment. Participants were required to walk on a 15 m walkway under a stable and comfortable speed, and five trials were recorded at baseline. All plantar loading data were processed with the Novel Multimask Evaluation software (Novel GmbH, Munich, Germany). The plantar foot was divided into seven regions in accordance with plantar anatomy, namely, heel (M1), midfoot (M2), the first metatarsophalangeal joint (M3), the 2nd and 3rd metatarsophalangeal joints (M4), the 4th and 5th metatarsophalangeal joints (M5), hallux (M6), and lessor toes (M7) with a mask (Figure 2) [32]. The data collectors were blind to the result of the random allocation sequence and were unclear about the aim of the study. The baseline data were collected in one week before the formal intervention, and the follow-up assessment was completed in one week after 6-month intervention.

### 2.6. Statistical Analysis

Independent *t*-test was used to analyse the differences among the characteristics (age, height, weight, and BMI), PP, and MF, and chi-square distribution was applied to compare the proportions of severity of knee at baseline of the Tai Chi group and the control group. The Shapiro–Wilk test was used for normal distribution to ensure that data distribution did not differ significantly from normal, and homoscedasticity was verified using Levene's test. A two-way ANOVA with repeated measure was used to examine the effects of interventions on plantar loads, including PP and MF of the whole foot and each region. Changes in outcome variables for participants in the Tai Chi and control groups were also determined with paired-samples *t* test, respectively. Analyses were performed using SPSS 20.0 software, and the significance level was set at 0.05.

## 3. Results

As shown in Figure 1, a total of 135 elders were recruited, and 89 elders were excluded. Among these individuals who completed the screening, 46 participants were randomly assigned into one of the two groups. Forty subjects completed the follow-up assessment (87%). Two participants in the Tai Chi group did not complete the study, due to time conflict and health-related issues, respectively. Four participants in the control group withdrew from the study, due to time conflict (*n* = 2) and unwillingness (*n* = 2). The characteristics of these subjects are listed in Table 1. No significant differences in demographic characteristics of participants were found between the Tai Chi group (age, 64.6 ± 3.4 years; body height, 154.8 ± 7.6 cm; body weight, 58.7 ± 8.3 kg; and BMI, 24.5 ± 3.kg/m21) and the control group (age, 64.5 ± 3.4 years; body height, 155.8 ± 4.8 cm; body weight, 62.8 ± 9.2 kg; and BMI, 25.9 ± 3.6 kg/m^2^) at the baseline. The PP and MF showed no differences between the Tai Chi group and the control group at baseline. Participants were also well-balanced between groups about scores of radiographic severity (Kellgren-Lawrence grade). Tables 2 and 3 illustrate the comparison for PP and MF of the Tai Chi group and the control group at the baseline and follow-up assessment.

### 3.1. Peak Pressure

There were statistically significant group × time interactions in total foot (*F* = 5.66, df = 1, *P* = 0.02), M1 (*F* = 10.35, df = 1, *P* = 0.01), and M4 (*F* = 14.90, df = 1, *P* = 0.01). Individuals receiving Tai Chi experienced no improvement in PP in total foot (*P* = 0.37), M1 (*P* = 0.14), and M4 (*P* = 0.08) between baseline and follow-up assessment. By contrast, those who received health program showed lower PP in total foot (*P* = 0.03), M1 (*P* = 0.01), and M4 (*P* = 0.01) at follow-up assessment. The statistically significant main effect of the within-group only showed in M5 (*F* = 7.08, df = 1, *P* = 0.01), which meant a greater PP in M5 after Tai Chi intervention.

### 3.2. Maximum Force

The ANOVA revealed significant group × time interactions for changes in M1 (*F* = 5.21, df = 1, *P* = 0.03) and M4 (*F* = 4.91, df = 1, *P* = 0.03). Individuals in the Tai Chi group also experienced no improvement in MF in M1 (*P* = 0.55) and M4 (*P* = 0.08) between baseline and follow-up assessment. By contrast, those who received health program showed lower MF in M1 (*P* = 0.02) at follow-up assessment except M4 (*P* = 0.21). The statistically significant main effect of the within-group only showed in M5 (*F* = 5.11, df = 1, *P* = 0.03), which meant a greater MF in M5 receiving Tai Chi intervention.

## 4. Discussion

Although Tai Chi exercise demands low posture [33], the ground contact loads of Tai Chi movements, such as Tai Chi gait [34] and push hand [35], are equal to the body weight of participants because these movements are gentle and fluid [33]. The present study investigated the effectiveness of Tai Chi in plantar loading for KOA individuals during walking after a 6-month intervention period. Results suggested that individuals in the Tai Chi group experienced larger PP and MF in 5th metatarsophalangeal joints and the control group had lower MF under heel and lower PP under total foot and the 2nd and 3rd metatarsophalangeal joints.

Our study showed that although a significant group × time interaction for the PP of the total region of foot was observed, there was no higher PP in the Tai Chi group and a lower PP in the control group. The significant change of whole foot PP of the control group in the present study was possibly due to that lower muscle strength in lower limbs. An experiment about the knee kinetics of individuals with KOA confirmed that they experience a significantly lower knee extensor moment than persons with no KOA [36]. Tai Chi practitioners had better isokinetic knee muscle strength than healthy participants [37]. Several studies also measured the kinematics of Tai Chi gait and normal gait and proved that Tai Chi gait exerts a large joint motion in ankle dorsiflexion, plantar flexion, knee flexion, and hip flexion [33, 38]. Meanwhile, an 8-week Tai Chi training could improve muscle strength of all lower limbs for community-dwelling participants aged 65 [39]. Therefore, Tai Chi intervention in our study would keep the muscle strength and PP for the total region.

Our study suggested that individuals receiving health program experienced lower MF, particularly on the rear foot, than individuals receiving Tai Chi exercise. Abnormally lower pressure on the heel for KOA participants is related to insufficient knee extension of individuals during the heel-contact phase than those of healthy elder persons [40]. A previous experiment about the knee kinetics of individuals with KOA confirmed that they experience a significantly lower knee extensor moment than persons with no KOA [36]. Furthermore, individuals with symptomatic KOA show significantly small knee range of motion during gait [41]. These changes in heel in our study implied that individuals in the Tai Chi group may maintain the biomechanics in the knee during walking after 6-month intervention because the program uses double-stance, weight-bearing, or single-stance weight-bearing movements from knee flexion to knee extension. The plantar loads of the control group were lower during the heel-contact phase than those at the baseline in our study, which demonstrated that individuals obtained low extension of the knee during walking.

In this study, the MF and PP were more significantly higher under M5 in the Tai Chi group and a lower PP in M4 was observed in the control group during walking. These changes might be related to the movement of Tai Chi. A previous study showed foot loads were mainly located in the anterior-medial areas in Tai Chi movements [32]. Moreover, most of the loads during the toe-off phase of gait are concentrated on metatarsal areas [42, 43] and increased forefoot load indicated an increased plantar flexion moment during walking [44]. A previous study also investigated the influence of regular Tai Chi practice on the muscle strength of the lower extremities in older people, and results demonstrated that the Tai Chi group generates more torque in their ankle dorsiflexion than the control group [45]. Meanwhile, the forefoot maintains the balance and cutaneous feedback during one-leg stance with eye open or closed [46]. The high plantar loading in the forefoot indicates a strategy so that elderly can maintain balance [47]. These findings may support the idea that Tai Chi can improve balance during walking, which is in agreement with those of previous studies demonstrating that Tai Chi reduces the risk of falling in older adult population [48, 49].

Although some studies implied that education program can provide benefits to cognitive ability and pain [50], the plantar loads in the control group of the present study showed a small decline after 6-month wellness education in our study. This decline might be related to intervention duration and frequency.

The current study also showed several potential limitations. First, the duration of Tai Chi might be not enough to show significant changes in all foot regions. Second, adding the individual weight may be considerably rigorous, although the weight in the current study showed no significant differences.

## 5. Conclusion

This study showed that these participants with KOA showed greater plantar loads in forefoot after 6-month Tai Chi intervention and Tai Chi could prevent the decrease of plantar loads during walking. Therefore, plantar load assessment would be a useful tool to assess the effect of Tai Chi on knee osteoarthritis.

## Figures and Tables

**Table 1:** Participants' characteristics at baseline.

Variable	Tai Chi (*n* = 23)	Control (*n* = 23)	*P* value
Age (y)	64.6 ± 3.4	64.54 ± 3.4	0.512
Body weight (kg)	58.7 ± 8.3	62.8 ± 9.2	0.191
Height (cm)	154.8 ± 7.6	155.8 ± 4.8	0.689
BMI (kg/m^2^)	24.5 ± 3.1	25.9 ± 3.6	0.242
Kellgren-Lawrence grade, *n* (%)			
1	7 (30)	6 (26)	
2	12 (52)	14 (61)	
3	4 (17)	3 (13)	

Data reported as mean ± standard deviation (SD). There were no significant differences between groups (*P* > 0.05).

**Table 2:** The peak pressure of the Tai Chi group and the control group at the baseline and follow-up assessment.

Region	Tai Chi		Control		Between-group	Within-group	Interaction
	Baseline	Follow-up	Baseline	Follow-up			
Total foot	345.81 ± 67.26	363.77 ± 88.77	354.14 ± 79.88	305.33 ± 79.88	0.261	0.278	**0.019**
M1	276.67 ± 60.64	298.52 ± 95.62	291.03 ± 65.74	244.28 ± 40.45	0.370	0.252	**0.006**
M2	173.26 ± 33.69	170.22 ± 34.06	176.91 ± 42.94	172.11 ± 24.43	0.144	0.270	0.091
M3	230.61 ± 67.96	246.45 ± 75.75	212.14 ± 81.24	200.03 ± 53.09	0.148	0.869	0.225
M4	280.46 ± 61.84	312.12 ± 70.61	319.19 ± 102.61	248.44 ± 50.01	0.573	0.151	**0.001**
M5	180.01 ± 55.45	214.12 ± 50.29	160.67 ± 47.87	158.01 ± 36.68	0.102	**0.012**	0.058
M6	242.55 ± 89.34	239.09 ± 69.18	230.36 ± 87.07	216.61 ± 44.06	0.452	0.518	0.698
M7	190.39 ± 53.41	179.39 ± 54.68	185.81 ± 52.11	158.28 ± 38.72	0.411	0.133	0.344

Values were means ± standard deviation (SD); significant differences (*P* < 0.05) are highlighted in bold. M1: heel; M2: midfoot; M3: the first metatarsophalangeal joint; M4: the 2nd and 3rd metatarsophalangeal joints; M5: 5th metatarsophalangeal joints; M6: hallux; M7: lessor toes.

**Table 3:** The maximum force of the Tai Chi group and the control group at the baseline and follow-up assessment.

Region	Tai Chi		Control		Between-group	Within-group	Interaction
	Baseline	Follow-up	Baseline	Follow-up			
Total foot	115.81 ± 15.73	119.29 ± 12.33	124.12 ± 17.89	114.65 ± 9.15	0.591	0.423	0.091
M1	70.05 ± 13.63	71.78 ± 13.54	68.88 ± 11.81	61.62 ± 7.53	0.143	0.171	**0.030**
M2	28.17 ± 6.77	27.51 ± 6.82	33.02 ± 5.07	28.89 ± 4.06	0.103	0.113	0.066
M3	22.81 ± 7.22	23.89 ± 8.32	18.31 ± 7.76	18.48 ± 7.82	0.073	0.257	0.417
M4	36.61 ± 7.62	40.41 ± 8.64	37.74 ± 10.51	34.68 ± 6.35	0.368	0.814	**0.034**
M5	15.84 ± 5.04	19.44 ± 5.82	14.11 ± 4.32	14.85 ± 4.62	0.071	**0.031**	0.190
M6	17.99 ± 6.67	18.49 ± 7.44	16.22 ± 6.71	15.64 ± 3.76	0.243	0.976	0.652
M7	25.24 ± 8.55	21.79 ± 8.18	21.88 ± 7.63	18.59 ± 4.57	0.169	0.109	0.947

Values were means ± standard deviation (SD); significant differences (*P* < 0.05) are highlighted in bold. M1: heel; M2: midfoot; M3: the first metatarsophalangeal joint; M4: the 2nd and 3rd metatarsophalangeal joints; M5: 5th metatarsophalangeal joints; M6: hallux; M7: lessor toes.

## Data Availability

The data used to support the findings of this study are available from the corresponding author upon request.
